# Patient decision making in recovering from surgery

**DOI:** 10.3389/fpsyg.2023.1170658

**Published:** 2023-06-20

**Authors:** Elizabeth Lerner Papautsky

**Affiliations:** Deptartment of Biomedical & Health Information Sciences, University of Illinois Chicago, Chicago, IL, United States

**Keywords:** patient ergonomics, human factors, patient work, naturalistic decision making (NDM), surgery, patient decision making

## Abstract

Patient work in surgery recovery is fraught with complex judgments and decisions. These decisions are not unlike ones that professionals make that we traditionally study with the Naturalistic Decision Making (NDM) theoretical lens and methods. Similarly, patients are making decisions in naturalistic settings and doing so with the objective of minimizing risk and maximizing safety. What is different is that patients are put in a position to perform complex, high level, high consequence work in the absence of any training, education, or decision support. Using a lived experience, I illustrate that the burden of judgement and decision making in surgery recovery work (e.g., caring for surgical sites, managing drains, managing medications, supporting activities of daily living) can be understood through a macrocognitive paradigm. Thus, the NDM theoretical lens and the associated methods is appropriate to study this problem space.

## 1. Introduction

Following discharge from the hospital and prior to their first follow-up visit, surgical patients face a challenging at-home recovery period – a time when complications may arise. The initial detection of these complications falls on the patient and thus, may be delayed or missed, posing a significant risk to clinical outcomes. One such example is the patient work associated with wound care and monitoring following surgery. Risks include surgical site infections (SSIs), at an annual cost of $3.3B in the United States (National Healthcare Safety Network, 2023). At rates ranging from 1% to 26% following breast surgeries due to breast cancer, SSIs are higher than the nationally reported incidence of what is expected for clean surgical procedures (Sattar et al., 2021). Post-discharge complications are higher with mastectomies (>100 K mastectomies annually in the United States) (Brigham and Women’s Hospital, 2023), with tissue that had been subjected to radiotherapy or chemotherapy (Bratzler and Houck, 2004; Palubicka et al., 2019) and in the presence of risk factors such as obesity, diabetes, and smoking (Sadok et al., 2022). In addition to the high financial cost of follow-up care and potential readmission (Yu et al., 2020), recovery may be filled with fear, uncertainty, and distress – impacting quality of life and potentially resulting in unfavorable patient outcomes (Larsson et al., 2022).

An interview study with 13 post-discharge surgical patients highlighted challenges in wound monitoring including lack of knowledge and self-efficacy, and accessible communication with their care team regarding their concerns (Sanger et al., 2014). There is limited literature on patient work of post-discharge care, suggesting a lack of recognition of this clinically relevant role. Further, there is a need to support patients in this role with education, training, and tools. Not just an opportunity to improve care quality and lower costs, effective patient support in post-discharge care has the potential to alleviate the cognitive (not to mention, the emotional) burden, improve patient safety, and move the needle towards patient-partnered care.

Despite countless calls to action, evaluation, and design work; it remains the case that discharge instructions are notoriously insufficient (Hoek et al., 2020; Cook et al., 2022). Practices for developing and delivering discharge instructions are inconsistent, resulting in variability across institutions, clinical settings, and procedure types. Frequently, patients are on their own to figure out what to do to care for themselves – resulting in developing strategies that may or may not be effective. In the best-case scenario, it is a struggle.

In terms of wound monitoring, the current standard of care is to instruct patients upon discharge to monitor for infection based on signs/symptoms of color, temperature, and tenderness (Sanger et al., 2014; Mousa et al., 2019). Visuals to help in making judgements regarding whether a wound warrants concern are unlikely to be included. However, alongside plenty of practical evidence, we have a robust literature spanning a multitude of domains including medicine, highlighting that humans with no training or experience perform poorly at identifying such vague and complex perceptual cues (Trueblood et al., 2018). Despite this, patients are in the position to make a judgement to identify a concern, as well as the subsequent decisions of if, when, and how to seek medical attention.

Several scientists have used the qualitative research method of autoethnography to reflect on experiences of their illness. According to Poulos (2021), autoethnography is grounded in self-reflexivity and draws upon analysis and interpretation of one’s lived experiences to characterize insights regarding the research problem space. Greenhalgh (2017) published an autoethnography of her experience of a 12-week course of adjuvant chemotherapy for early stage breast cancer. A social scientist and a doctor herself, Greenhalgh’s account differs from many other cancer stories, in that it is anchored in medical evidence. Greenhalgh’s favored definition is: “writing about one’s own experiences for specific academic purposes” (Richards, 2008, p. 1718). A patient is in a unique position to conduct such an investigation as they are the only one who is privy to observing and capturing the full continuum of their experience. When such work is conducted by a scholar of system science, social science, medicine (or other related disciplines); as a function of continuity, they have the opportunity to identify the gaps. These gaps may not come to light through research that is based on sampling. For instance, ethnographic observations capture ranges or multiple points in time, but do not capture the full care continuum. One might argue against putting too much emphasis on such accounts due to bias. However, no research approach is devoid of bias. To improve patient safety requires a shift in how we think, talk, and approach the study of patient experience.

In this paper, by presenting an autoethnography of at-home post-discharge surgery recovery, I illustrate that the burden of judgement and decision making based on complex cognitive and perceptual cues falls on the patient. I argue that we must begin to recognize that patients conduct work in the context of uncertainty, dynamic circumstances, vague goals, time stress, multiple players – characteristics highlighted by Klein (2008) as ones that define decision making in complex environments. Similarly, as knowledge of professionals (e.g., soldiers, nuclear power plant operators, doctors, nurses) must be acquired via training and assessed, patient knowledge matters too. In the context of chronic illness, some patients develop deep knowledge about their illness and its management. Conversely, most patients have no knowledge or experience to apply to the required work of at-home post-discharge recovery – they are novices. Given that Naturalistic Decision Making (NDM) (Klein, 2008) is concerned with complex decision making in real-world situations, with particular focus on capturing expertise and delivering it to novices, this theoretical lens is appropriate for characterizing patient decision making.

## 2. Overview of surgery, time in the hospital, and guidance for recovery

In 2019, as part of early stage breast cancer treatment, I had a double skin-sparing mastectomy with an immediate deep inferior epigastric perforator (DIEP) flap reconstruction. A double skin-sparing mastectomy is the removal of both breasts, while leaving most of the skin intact for reconstruction (American Cancer Society, 2021a). A mastectomy is conducted by a breast surgeon. Mine took approximately 5 h. A DIEP flap reconstruction is the rebuilding of the breasts using one’s own tissue. Specifically, the skin, fat, and blood vessels are taken from the lower abdomen and used to reconstruct the breasts (American Cancer Society, 2021b). It is a microsurgery, where the tiny blood vessels in the abdominal tissue are matched to blood vessels in the chest and reattached under a microscope. It is conducted by a plastic surgeon and varies in the number of hours that it takes. My mastectomy and reconstruction was a single surgery that lasted 12 h and was followed by 3-night hospital stay at an urban academic medical center. This length of stay is covered by insurance in the United States healthcare system, as a best-case scenario – in cases with no immediate complications.

I have limited memory of the hospital stay because I was medicated and slept the majority of the time. At least twice, I participated in physical therapy – I was taught how to sit up and get out of bed and was taken on a walk in the corridor outside of my room. My mother was present throughout the entirety of my hospital stay. She spent nights on a fold-out bed in my hospital room. She took opportunities to observe the work of the clinical care team members. She watched the nurse and asked questions about managing drains and logging their output. She observed the surgical team check my surgical sites during their rounds and was thus, aware of their appearance. On the day prior to discharge, she participated in helping the nurse prepare me for a shower by securing my drains, helping me out of bed, and transferring me into the shower. At discharge, she received a printed package of instructions that included guidance on drain and medication management, along with multiple prescriptions to be picked up at the pharmacy.

The recommended recovery time for a DIEP flap reconstruction is 6–8 weeks (BreastCancer.org, 2022), with the first 2 weeks being the most challenging due to risks of complications, physical limitations and cognitive impairment. Per surgeon instructions, I was not allowed to stand up straight for 2 weeks due to the incision that ran horizontally from hipbone to hipbone. Hunching over resulted in back pain while walking, following by a persistent ache that was most bothersome at night. Other physical impairments included limited range of motion due to sutures and pain at surgical sites, inability to engage in strenuous activities, and swelling in extremities due to fluid retention. I also struggled with freely navigating my physical environment due to the potential of my 4 surgical drains (that either hung, were secured, or needed to be carried) getting caught or snagged, which was painful and I constantly worried that they would get pulled out. On the DIEP flap Facebook group that I relied upon for surgery preparation and recovery strategies, patients consistently reported surgical drains as being the “worst” part of the surgery. Factors responsible for my impaired cognitive state included the side effects of pain management medications (e.g., opioids), having undergone full anaesthesia for the duration of a lengthy surgery, and psychological distress associated with surgery and recovery. Cancer-related cognitive impairement is a side effect that is becoming more recognized than it had been in the past (Van Dyk and Ganz, 2021).

**Figure 1:**
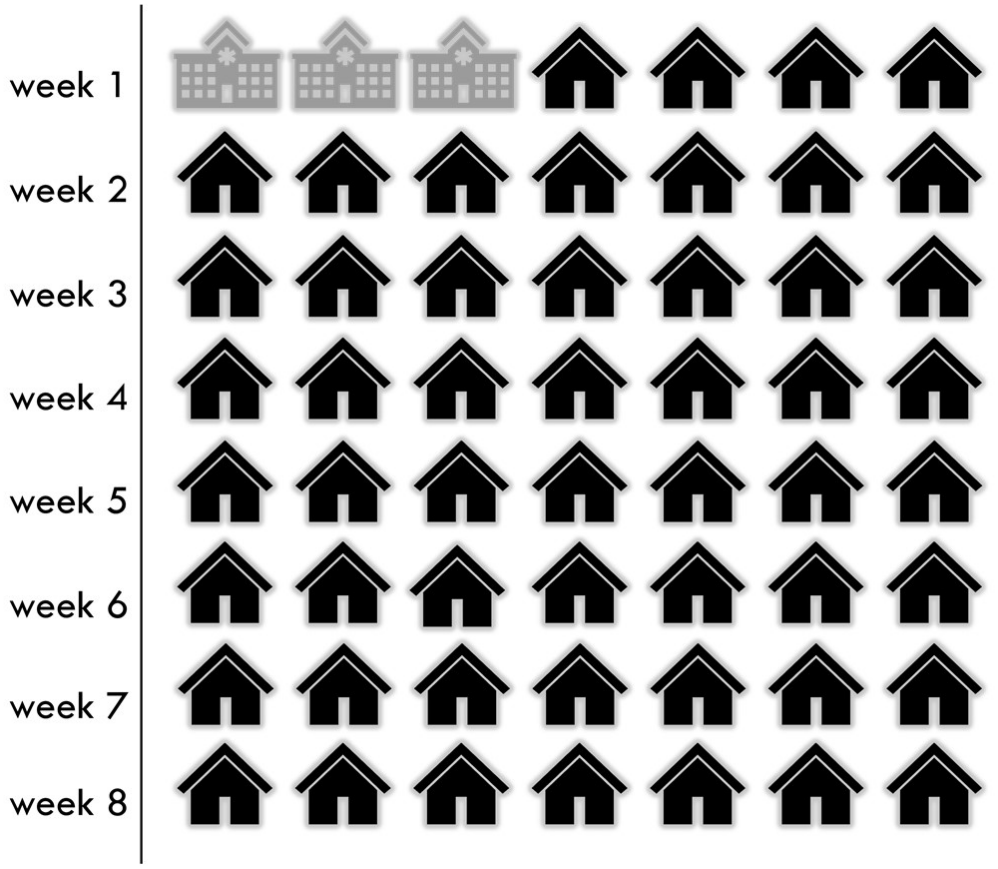
Days spent in hospital versus at home during the recovery period.

Coming home only 3 days into my recovery was difficult. In Figure 1, I represent the time spent in the hospital under the supervision of healthcare workers versus the at-home recovery under the supervision of self and caregiver with no clinical knoweldge or experience, over the recommended recovery timeline.

## 3. Patient work

In recent years, there has been an increasing amount of focus on patient work (Holden and Valdez, 2021). There is now recognition that patients conduct not just work that is visible (tangible, observable), but also work that is invisible because it is cognitive or perceptual. A 2020 scoping review proposes several categories of visible work that vary based on whether it is conducted individually or in collaboration and consumes or creates resources (or both) (Yin et al., 2020). Authors highlight the importance of effective task performance and provide examples such as management of medications and physical environment as examples. Invisible work, on the other hand, is work that is either unseen or undervalued (Gorman et al., 2018) and may be cognitive (Yin et al., 2020) or perceptual in nature. Invisible work remains privileged, unless revealed by the patient. The work of DIEP flap recovery included both visible (task-oriented, procedural) and invisible (cognitive and perceptual).

We (myself and my caregiver) performed procedural, stepwise tasks including the management of 9 surgical sites and 6 medications. The surgical sites included: (1) left breast, (2) right breast, (3) abdomen (scar from hipbone to hipbone), (4) belly button (reconstructed), (5) right under arm (due to axillary node removal), and (6–9) 4 surgical drains (1 at each breast, 1 at each side of abdomen). Several of the sites were multiple inches in length. The medications included: (1) pain reliever (e.g., opioid, then Acetaminophen), (2) blood thinner (self-administered sub-cutaneous injection), (3) anti-nausea medication, (4) antibiotic, (5) stool softener, and (6) others (e.g., benzodiazepine for anxiety). At least 2 of the medications contributed to cognitive impairment and had the potential to be addictive.

More complex work involved management, identification, and mitigation of risk – with implications for outcomes. Implications were a function of effective task performance that included interpretation of complex perceptual cues. Specifically, we had to actively manage infection control, fall prevention, medication safety, identification of blood clots, and identification of potential infections. All of these were iterative processes comprised of decision points. For example, we monitored changes to wound appearance as a function of time – e.g. *wound looks different this morning from the way it looked last night – it looks more/less red*. If something appeared concerning, we made a judgment on whether to contact the clinical care team immediately or to wait – a decision point. The consistent challenge was that we did not know *what the wound was supposed to look like*. Additionally challenging was that given the nerves were severed in the breasts and the abdomen, I had no sensation and could not rely on symptoms such as pain or warmth for these potentially critical judgments. When we did contact the clinical care team, the narrative and visual information that we shared may have been incomplete, inadequately described (given our lack of knowledge of technical or descriptive terminology), and potentially unreliable. To be sure, remote SSI surveillance is challenging, resource intensive, and error prone (Macefield et al., 2023), partly because patient-taken photographs are unreliable given inconsistent lighting, angle, resolution, and skill. A 2019 study examining wound photography to remotely assess SSIs by 523 surgeons found that although photographs increased confidence, they decreased detection sensitivity (Kummerow Broman et al., 2019). This evidence suggests that there is a need for patients to advocate for themselves further if they are concerned about a potential infection.

In Table 1, I identify the patient work (caring for surgical sites, managing drains, managing medications, supporting activities of daily living) associated with risk management and mitigation, resources involved, along with a description of challenges or implications. All described processes rely upon knowledge, comprehension, and strategies and/or support tools (potentially developed by oneself).

**Table 1:** Patient surgery recovery work characterization.

	Tasks	Tools & artifacts	Risk mitigation
Caring for surgical sites	• Cleaning	• Wipes	Infection control: conducting wound care with no prior experience or formal medical training. Aside from the technical aspects of this work, it is critical to be aware of and follow precautions associated with infection control including hand washing, using antiseptic wipes, and even showering and changing clothes on a regular basis. Type: procedural
	• Changing bandages	• Bandages	
	• Monitoring for changes	• Photos	
Managing 4 drains	• Emptying		Infection monitoring & blood clot monitoring: monitoring and making judgements based on perceptual cues associated with infection and blood clots is complex. For instance, cues including color, temperature, feel, size, and change perception are associated with monitoring for infection. They are difficult to interpret if lacking clinical knowledge and experience. Type: cognitive and perceptual
	• Monitoring and documenting color, consistency, and amount of fluid output	• Drain log, pen	
Managing medications	• Following and resolving discharge instructions with instructions printed on the pill bottles	• Discharge instructions, pill bottle instructions	Medication safety: managing multiple medications on a schedule is a cognitively complex task that relies on attention, memory, as well as development and use of artifacts (e.g. Logs) as memory aids. Effective tools and strategies are critical. Type: procedural and cognitive
	• Following a schedule	• Alarm	
	• Documenting	• Medication log, pen	
Supporting activities of daily living	• Walking	• Walker	Fall prevention: setting up the physical environment to accommodate temporary physical limitations, as well as physical support and vigilance (e.g. Physical presence, auditory access, allocation of attention) from the caregiver(s). Physical supports (considerations for design, availability, placement) for bathing and toileting play a role in reducing fall risk. Type: ergonomic and cognitive
	• Bathing	• Grab bar, shower chair, safety pins for drains	
	• Toileting	• Raised toilet seat, bidet	
	• Feeding	• Hospital table	

Further, I highlight that although infection control may be mainly visible (e.g., washing hands, using alcohol wipes), monitoring for infection is more complex. Specifically, it involves making judgements based upon complex cognitive and perceptual cues. Cues such as color, temperature, feel, size, and change perception are associated with monitoring for infection. The interpretation of such cues is particularly challenging if lacking clinical knowledge and experience and is thus prone to error (miss or delay of infection identification). Decisions made based on these judgments – if, when, how, to whom to report concerns by calling, emailing, and/or sending photos of surgical sites – have potential implications for safety and outcomes such a delay in diagnosis of an infection requiring the need for follow-up care or hospitalization. These judgements are consistent with macrocognitive processes executed by professionals in complex settings described by Klein (2008) – e.g. *discovering*, *detecting problems*, *sensemaking*, managing uncertainty, *planning*, *deciding-acting*.

## 4. Implications for solutions

Surgical patients play a clinically relevant role in post-discharge recovery. Given their lack of knowledge, they are vulnerable to delaying seeking clinical support with the potential of negative clinical consequences. Therefore, there is an urgent need to characterize the depth and complexity of their cognitive and perceptual work. This is the only way to make progress towards effective patient-facing support solutions. NDM was specifically developed to capture state of knowledge and comprehension of humans performing in complex real-world settings. Thus, it is appropriate to apply NDM (a theoretical lens coupled with elicitation methods of Cognitive Task Analysis (CTA), and specifically, Critical Decision Method (CDM)), to characterize patient work. Further, findings are critical in informing the development and evaluation of patient-centered support solutions ranging from effective discharge instructions to education and decision aids on identifying infections and blood clots in real-time.

## 5. Conclusion

Patient work is fraught with complex judgments and decisions. These decisions are not unlike ones that professionals make that we traditionally study with an NDM theoretical lens and methods. Similarly, patients are making decisions in naturalistic settings – in the home or other contexts of daily living. Moreover, not unlike professionals, they are performing with the objective of minimizing risk and maximizing safety. They are in fact, the biggest stakeholder. What is different is that patients are put in a position to perform complex, high level, high consequence work in the absence of any training, education, or decision support. The reason for this largely invisible travesty is financial, rather than patient safety. The solution lies with developing effective patient support informed by understanding of their work. However, a deep understanding of the patient as a decision maker continues to remain a research and operational gap. NDM is a promising toolkit to adopt and adapt to make progress towards filling this gap.

## Data availability statement

The original contributions presented in the study are included in the article/supplementary material, further inquiries can be directed to the corresponding author.

## Ethics statement

Ethical review and approval was not required for the study of human participants in accordance with the local legislation and institutional requirements. Written informed consent from the patients/participants or patients/participants legal guardian/next of kin was not required to participate in this study in accordance with the national legislation and the institutional requirements.

## Author contributions

The author confirms being the sole contributor of this work and has approved it for publication.

## Conflict of interest

The author declares that the research was conducted in the absence of any commercial or financial relationships that could be construed as a potential conflict of interest.

## Publisher’s note

All claims expressed in this article are solely those of the authors and do not necessarily represent those of their affiliated organizations, or those of the publisher, the editors and the reviewers. Any product that may be evaluated in this article, or claim that may be made by its manufacturer, is not guaranteed or endorsed by the publisher.
